# What is the airspeed velocity of an unladen swallow? modeling numerical judgments of realistic stimuli

**DOI:** 10.3758/s13423-023-02331-0

**Published:** 2023-10-06

**Authors:** David Izydorczyk, Arndt Bröder

**Affiliations:** Department of Psychology, School of Social Sciences, University of Mannheim, Mannheim, Germany

**Keywords:** Numerical judgments, Cognitive modeling, Multidimensional scaling, Natural stimuli

## Abstract

Research on processes of multiple-cue judgments usually uses artificial stimuli with predefined cue structures, such as artificial bugs with four binary features like back color, belly color, gland size, and spot shape. One reason for using artifical stimuli is that the cognitive models used in this area need known cues and cue values. This limitation makes it difficult to apply the models to research questions with complex naturalistic stimuli with unknown cue structure. In two studies, building on early categorization research, we demonstrate how cues and cue values of complex naturalistic stimuli can be extracted from pairwise similarity ratings with a multidimensional scaling analysis. These extracted cues can then be used in a state-of-the-art hierarchical Bayesian model of numerical judgments. In the first study, we show that predefined cue structures of artificial stimuli are well recovered by an MDS analysis of similarity judgments and that using these MDS-based attributes as cues in a cognitive model of judgment data from an existing experiment leads to the same inferences as when the original cue values were used. In the second study, we use the same procedure to replicate previous findings from multiple-cue judgment literature using complex naturalistic stimuli.

## Introduction

**Bedevere**: “How do you know so much about swallows?” **Arthur**: “Well, you have to know these things when you’re a king, you know.” in *Monty Python and the Holy Grail* (Jones & Gilliam, 1975)

Imagine yourself in the of role of Arthur, King of the Britons in the movie *Monty Python and the Holy Grail* (Jones & Gilliam, 1975). Wanting to cross the Bridge of Death you have to answer the evil bridgekeeper’s question: “What is the airspeed velocity of an unladen swallow?”. Assuming you are unaware of the difference between African and European swallows and don’t know the actual airspeed velocity of around 50 km/h (Park, Rosén, & Hedenström, 2001), you would have to give a numerical estimate. Such numerical estimates or judgments are not exclusive to natural domains, such as birds, but are an integral cognitive activity which guides and informs our behavior in all areas of our life.

For many years, researchers investigated the cognitive processes underlying numerical judgments, developed formalized computational models which capture these processes and tested what influence the environment, the learning history, different cognitive systems or abilities have on these processes (e.g., Brehmer , 1994; Bröder & Gräf , 2018; Brunswik , 1955; Hoffmann, von Helversen, & Rieskamp , 2013; Juslin, Olsson, & Olsson , 2003; Mata, von Helversen, Karlsson, & Cüpper, 2012; Pachur & Olsson, 2012; Persson & Rieskamp , 2009; von Helversen & Rieskamp , 2009). Two of the most prominent cognitive process models describing multiple-cue judgment processes are *rule* and *exemplar* models. In exemplar models, the judgment of a given object is generated by retrieving similar objects from long-term memory and by forming a similarity-weighted average of their criterion values (e.g., Izydorczyk, & Bröder , 2021; Juslin, Olsson, & Olsson, 2003; Medin & Schaffer , 1978; Nosofsky , 1984). In rule-based models, the judgments rely on abstract knowledge about cue-criterion relations, such as a linear combination of cues (e.g., Brehmer , 1994; Einhorn, Kleinmuntz & Kleinmuntz , 1979; Juslin, Olsson, & Olsson, 2003). Based on empirical findings, more recent models, such as the RulEx-J model (Bröder, Gräf, & Kieslich, 2017) or the CX-COM Model (Albrecht, Hoffmann, Pleskac, Rieskamp, & von Helversen, 2019), assume some form of mixture between both types of processes (see also, Herzog, & von Helversen , 2018; Hoffmann, von Helversen, & Rieskamp , 2014; Wirebring, Stillesjö, Eriksson, Juslin, & Nyberg , 2018).

However, these cognitive models have only been tested in experiments which use artificial stimuli varying along a small number of dimensions. For instance, fictitious bugs varying on four binary cues (e.g., Juslin, Karlsson, & Olsson , 2008; Trippas & Pachur, 2019), patients suffering from a fictitious tropical disease with four distinct symptoms as cues (e.g., Persson & Rieskamp , 2009; Platzer & Bröder , 2013), comic figures with four possible binary cues (e.g., Hoffmann, von Helversen, & Rieskamp, 2014), or fictitious job candidates with different binary skills (e.g., knowledge of French or Italian, Scholz, Helversen, von, & Rieskamp , 2015; von Helversen, Herzog, & Rieskamp , 2014). The reason for this, beside experimental control, is that all computational models rely on identifiable cues or attributes of the judgment objects that form the basis of the computations. In exemplar-based models, the cues are needed to compute the similarity between exemplars, which are the basis of the resulting judgment (Juslin, Olsson, & Olsson, 2003). In rule-based models, the cues are combined directly to produce a judgment (Brehmer , 1994; Einhorn, Kleinmuntz & Kleinmuntz, 1979; Juslin, Olsson, & Olsson, 2003). However, for complex, natural, real-world objects the cues or features people use to represent these stimuli in memory and base their judgment on, are rarely known. This makes it difficult to apply the cognitive models and corresponding findings to real-world domains.

Building on the early classification and categorization literature, in this article we demonstrate in two studies how to combine *multidimensional scaling* analysis (MDS, Hout, Papesh, & Goldinger , 2013; Kruskal , 1964; Shepard , 1962) with a current state-of-the-art model for quantitative judgments to investigate the underlying processes of judgments of complex, natural stimuli where the cues are not known beforehand. We will use the cues extracted by a MDS to model data with the *RulEx-J* model (Bröder, Gräf, & Kieslich, 2017; Izydorczyk, & Bröder , 2022) which measures the relative contribution of rule- and exemplar-based processes. We will use different kinds of stimuli (simple artificial vs. complex and naturalistic) and manipulate different learning regimes (different information during judgment task vs. different tasks during learning) to affect the kind of processing people predominantly use.

### Related research on extracting and generating cues

The procedure we present here originates from early research using the *generalized context model* (GCM, Nosofsky , 1984; Nosofsky , 2011) to describe exemplar-based categorization processes. According to the GCM, classification decisions are based on the summed similarity of a to-be-judged item (i.e., the probe) to the exemplars of one category relative to the exemplars of alternative categories. The GCM uses a similar approach like MDS to model the similarity exemplars and the probe, where exemplars are represented as points in a multidimensional psychological space, and the similarity between each exemplar and the probe is a decreasing function of their distance in this space (Nosofsky, 1986, 1992, 2011; Shepard, 1957, 1962, 1987). In order to apply the GCM, the cues and cue values, which define the location of the exemplars in space, where often derived by using a MDS study beforehand (Nosofsky, 1992). For instance, Shin and Nosofsky (1992) derived an MDS solution for different dot patterns based on similarity ratings between these dot patters. The MDS solution was then used as basis in mathematical prototype and exemplar models for predicting classification and recognition data. A similar approach was taken in a recent series of studies by Nosofsky and colleagues, where they used similarity judgments of different minerals to derive MDS-based cue dimensions which then served as a basis for the cognitive modeling of people’s subsequent categorizations (Nosofsky, Sanders, Meagher, & Douglas, 2018, 2020; Nosofsky, Sanders, & McDaniel, 2018; Nosofsky, Sanders, Zhu, & McDaniel, 2019). This differs from the approach taken in multiple-cue judgment studies, where the cues and cue values of exemplars and stimuli are predefined by the experimenters (e.g., Hoffmann, von Helversen, & Rieskamp, 2014; Trippas & Pachur, 2019). For instance, Juslin, Olsson, and Olsson (2003) presented participants with bugs differing in four binary visual cues (e.g., length of legs, color of the back). Since the cues were binary, they could take values of 1 or 0. Each fictitious bug was then represented by a four dimensional 0/1 vector (e.g., [0, 1, 0, 1]). This approach, however, would be not feasible if the to-be-judged stimuli were not designed by the experimenter or the cues and cue values were unknown. This makes it difficult to transfer and test important experimental findings of multiple-cue judgment experiments to situations with more realistic stimuli or with an applied focus (Goldstein & Hogarth, 1997).

Instead of using MDS analysis to generate (low) dimensional representations of stimuli based on similarity ratings, recent studies used deep neural networks (DNN) to generate high-dimensional representations of natural images or words (e.g., Günther, Rinaldi, & Marelli , 2019; Roads & Mozer , 2021; Zou & Bhatia , 2021; for an overview see Bhatia & Aka, 2022). In these studies, researchers extracted

feature representations from pre-trained DNN for classifying images or predicting word coocurrences. For instance, Peterson, Abbott and Griffiths (2018) extracted 4,096-dimensional vectors from the final hidden layer of a highly popular image classifying DNN (VGG, Simonyan & Zisserman , 2014), to get numerical representations of given images. Several studies with similar procedures have shown that these high-dimensional representations can be used in categorization models (Battleday, Peterson, & Griffiths, 2020), to predict human similarity ratings (e.g., Peterson, Abbott, & Griffiths , 2018; Roads & Mozer , 2021), or other continuous judgments, such as masculinity or femininity of words (Richie, White, Bhatia, & Hout, 2020), or the calory content of different foods (Zou & Bhatia, 2021). Although, DNNs are becoming increasingly popular for extracting feature representations, in this study we will rely on the traditional (and in our opinion better) approach of collecting pairwise similarity ratings and subsequently conducting MDS analysis.^1^

However, so far, none of these works combined these methods for generating cues for complex real-world stimuli^2^ with state-of-the-art models of the multiple-cue judgment literature and none investigated whether classic findings from the laboratory can be replicated with non-artificial, naturalistic stimuli. In this article, we want to close this gap in the literature.

### Aims and outline of this article

**Fig. 1:**
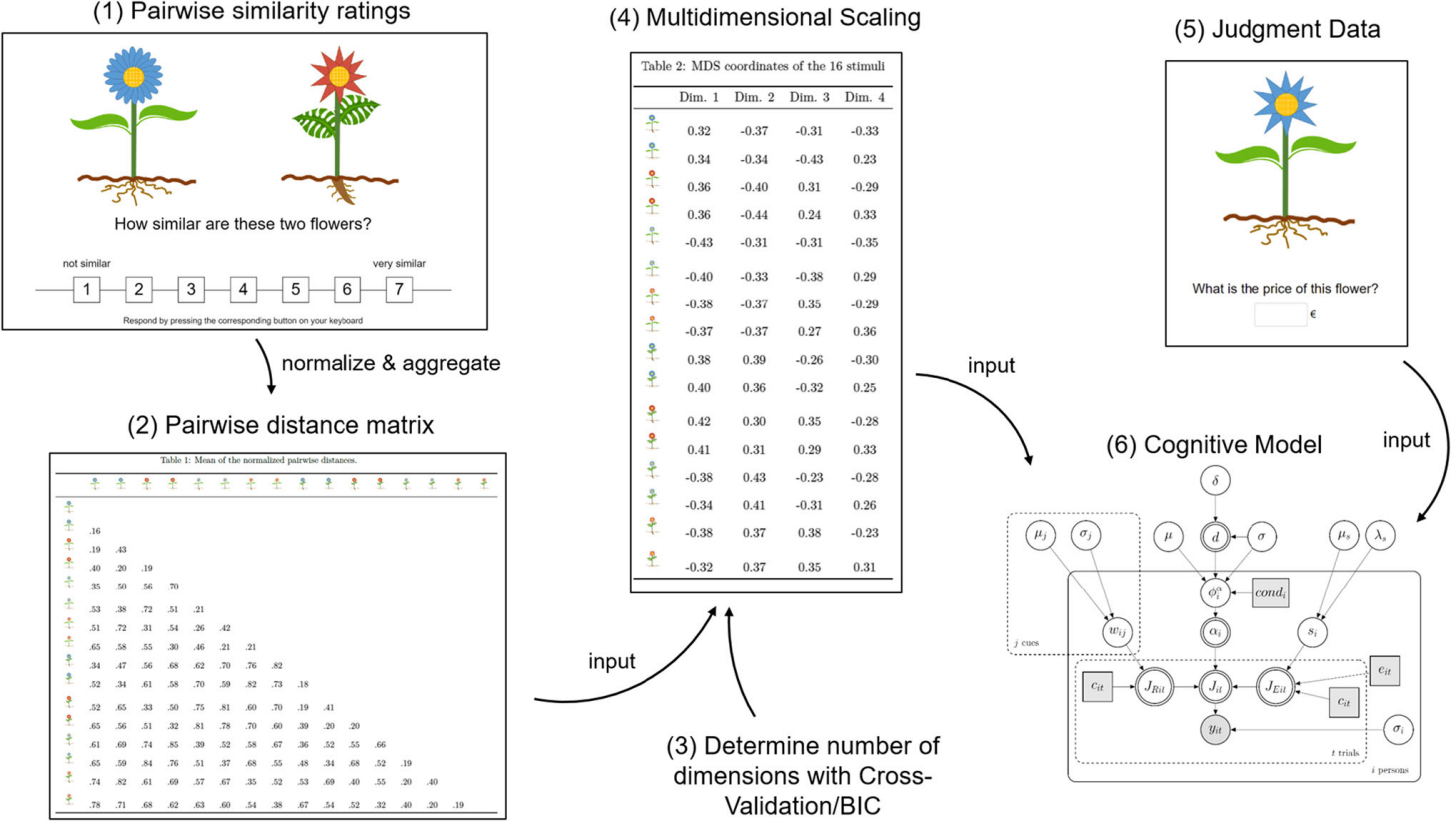
This figure depicts the general procedure used in Studies 1 and 2. Pairwise similarity ratings from a norming sample are collected as data (1) which are transformed to pairwise distances (2). From these, an MDS (4) extracts dimensions, the number of which is determined by BIC or cross-validation (3). Finally, a computational model (6) can be applied to judgment data (5), using the MDS dimensions as cues. The flowers are examples of stimuli used in Izydorczyk and Bröder (2022)

In this work, we present two studies. Still using artificially created simple stimuli, the validation study serves as a proof-of-concept demonstrating that (a) existing (and known) cue structures are well recovered by an MDS analysis of similarity judgments and (b) that using the MDS-based attributes as cues in a cognitive model of judgment data from an existing experiment leads to similar predictions and inferences as when the original cue values were used. The second study extends the general workflow from the validation study to natural stimuli, in which case no cues are known beforehand, and tries to replicate classic multiple-cue judgment findings using complex, real-world stimuli, namely bird species. Each step of this workflow and how they relate is shown in Fig. 1. All analysis were conducted using R Version 4.2.2 (R Core Team, 2022). The Bayesian models were implemented with JAGS Version 4.3.0 (Plummer, 2003). All experiments were run online using lab.js (Henninger, Shevchenko, Mertens, Kieslich & Hilbig, 2022).

## Study 1: Validation

The goal of the first study was to validate the general procedure shown in Fig. 1 by investigating whether the experimentally created attribute structure of artificial stimuli would be recovered by an MDS analysis (e.g., Nosofsky , 1989) and more importantly, whether the cues extracted from the MDS analysis could be used to model empirical judgment data. The judgment data we used for this purpose are data from the validation experiment reported in Izydorczyk and Bröder (2022),^3^ where $n$ = 238 participants judged the selling price of 16 fictitious flowers that varied on four binary attributes (see Fig. 1 for some examples). Participants got different aiding information and instructions to solve the task, which, depending on the condition, should lead to either more rule- or more exemplar-based processing. By using data and artificial stimuli with an experimentally created attribute structure of an existing experiment, the first study was designed to test the general idea of this paper (i.e., using MDS-based attributes as a basis for cognitive modeling of quantitative judgments), as well as the different steps of our analysis pipeline (i.e., procedure for determining the number of MDS dimensions, etc.). For reasons of brevity, we will only summarize the main results of the validation study here. A detailed description of the experiments, analysis and results can be found in the supplementary materials on the OSF.

### Results summary

The findings indicated that the MDS analysis accurately captured the attribute structure of the simple artificially created stimuli based on the pairwise similarity ratings of $N$ = 40 participants, consistent with several prior studies (e.g., Nosofsky , 1989; Nosofsky , 1991). By employing a cross-validation procedure, we determined that a four-dimensional MDS-space best described the pairwise similarity ratings, and that these dimensions corresponded perfectly to the four actual attributes of the stimuli, as indicated also by the very high correlation between the predicted and empirical pairwise distances ($r(118) =.99, p <.001$).

Furthermore, using the derived MDS attributes as cues in the hierarchical Bayesian RulEx-J model (see the next section for details) yielded the same conclusion as when the experimentally created attributes were used as cues: The model indicated more rule-based processing in the condition supposedly inducing this kind of process and more exemplar-based processing in the exemplar condition.

Thus, the validation study demonstrated the general applicability of our procedure to model judgments of stimuli with unknown cue-structure.

## Study 2: Application to naturalistic stimuli

The validation study showed that the cue structure of artificial stimuli can be recovered by an MDS analysis based on pairwise similarity ratings (see also, Nosofsky , 1989; Nosofsky , 1991), which, in turn, allowed to model the numerical judgments of participants. In this second study, we test whether this result extends to non-artificial stimuli with an unknown cue structure. For this purpose, using images of birds, we plan to replicate the robust finding from the multiple-cue judgment literature that the type of learning task and feedback impacts the strategy selection in subsequent judgment tasks (Pachur & Olsson, 2012; Trippas & Pachur, 2019). In these studies, participants who had to compare two stimuli in the trials of the training phase and only received feedback whether their choice was correct (learning by comparison) showed more rule-based processing and overall better generalization ability (i.e., higher accuracy when estimating new stimuli), than participants who were presented with only one stimulus at a time but who received feedback about the actual criterion value (direct criterion learning). Based on the results of three experiments, Trippas and Pachur (2019) suggest that the greater reliance of rule-based strategies in the learning by comparison condition is mainly due to the relative feedback provided during trials and also due to the missing continuous criterion information, which is needed for exemplar-based judgment processes.

**Fig. 2:**
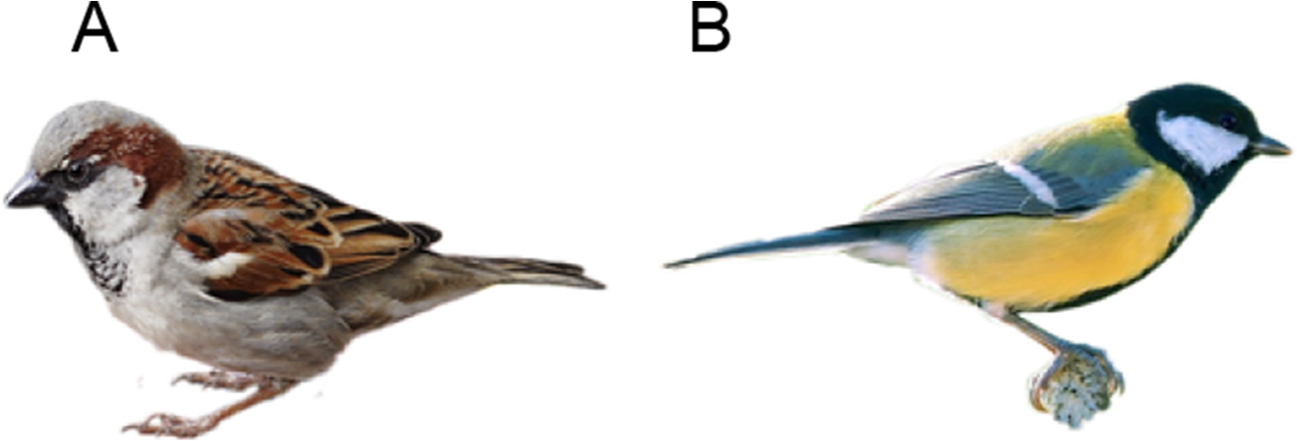
Example of stimuli used in Study 2. **A** House sparrow (lat. Passer domesticus). **B** Great tit (lat. Parus major). Images are available under a CC BY-SA 4.0 license on the OSF

The overall procedure in this second study was the same as in the validation study and as shown in Fig. 1. We first generated cues for each stimulus, based on pairwise similarity ratings and a subsequent MDS analysis (Steps 1 to 4 in Fig. 1). We then used the generated cues in the hierarchical Bayesian RulEx-J model to analyze the data from a preregistered experiment whose procedure was based on Pachur and Olsson (2012) and Trippas and Pachur (2019) (Steps 5 and 6 in Fig. 1).

### Similarity ratings & MDS analysis (Steps 1 to 4 in Fig. 1)

#### Method

##### Materials

The stimuli were 32 images from different common and generally known birds. The selection of birds was based on a quartets card game. For each bird, we selected a high-quality image based on a Google image search, showing the bird in a sitting position. We used photo-editing procedures to remove the background and to rescale the images to a size of 250px by 250px. Figure 2 shows two examples of the final stimuli. The complete list of stimuli as well as the corresponding images can be found at the OSF.^4^

##### Design and procedure

After giving informed consent, participants were instructed that we were interested in how people judge the similarity of birds. To reduce the burden on participants, a balanced incomplete block design was used in which each participant rated only a sample of $k$ = 124 bird pairs out of a total all possible $K$ = 496 stimulus pairs. To ensure that each bird pair received a sufficient number of ratings, every group of four participants rated all 496 pairs, but the assignment of which bird pairs each participant rated was randomized. Similarity ratings were provided on a scale ranging from 1 (not similar) to 7 (very similar). With this procedure, each bird pair was rated on average by 24.25 ($SD$ = 2.15) participants. In every trial, a randomly selected pair of birds was shown to the participants in the center of the screen (Step 1 in Fig. 1). The order of the stimulus pairs, as well as the location of the individual birds of each pair on the screen (e.g., left or right side) was randomized. At the end of the experiment, participants answered demographic questions and were asked to indicate if their data should be used for data analysis or if it should be excluded (Aust, Diedenhofen, Ullrich, & Musch, 2013).

##### Participants

In total, we collected data from $N$ = 110 participants through Prolific Academic. We excluded $n$ = 3 participants because they indicated that their data should not be used for analysis and $n = 10$ participants because they finished the complete survey in less than three minutes.^5^ Thus, the final sample consisted of $N$ = 97 participants (64.95 % female) with an average age of 35.37 ($SD$ = 12.85).

##### Data analysis

For the multidimensional scaling analysis, we averaged the similarity judgments for each stimulus pair across participants. We then normalized the average similarity judgments for each pair, so that they ranged from 0 to 1 and then subtracted these normalized similarities from one to yield dissimilarities^6^ (Step 2 in Fig. 1). The resulting normalized average dissimilarity matrix was then subjected to a non-metric multidimensional scaling analysis using the smacoef package (Mair, Groenen, & de Leeuw, 2022) in R (Step 4 in Fig. 1).

In order to perform an MDS analysis, one has to specify the number of dimensions the MDS solution should have (Step 3 in Fig. 1). Based on the results of the validation study, we decided to use a cross-validation approach (Hastie, Tibshirani, & Friedman, 2009; Richie, White, Bhatia, & Hout, 2020; Steyvers, 2006) to determine the number of dimensions of the resulting MDS solution. In one iteration of the cross-validation, for one specific number of dimensions, we randomly removed 20% of the entries from the aggregated normalized pairwise distance matrix. We then fitted an MDS solution to this reduced matrix. Next, we predicted the pairwise distances for the hold-out cells based on the resulting MDS solution. The cross-validation criterion was then the average correlation between the predicted and the true pairwise distances over 500 repetitions.^7^

**Fig. 3:**
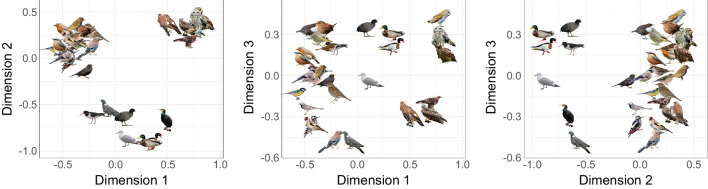
Results of the three-dimensional solution from the multidimensional-scaling analysis of the pairwise similarity ratings of 32 bird images. However, interpreting the dimension is not straightforward. Dimension 1 might correspond to the size of the birds. Dimension 2 separates between water and ’land’ birds (except the dove). Dimension 3 might be related to shape (round vs. slim) of the birds. Axis scales sometimes differ in order to allow better visualization of the bird pictures.

#### Results

The results of the cross-validation procedure are shown in Table 1. According to the cross-validation procedure, the best number of dimensions to use for the MDS analysis was three ($\bar{r}$ =.88). The correlation between the observed and predicted pairwise distances was again very high, $r(494) =.93, p <.001$. The resulting configuration of all 32 birds based on the MDS solution with three dimensions is shown in Fig. 3.

**Table 1:** Indices to determine the number of dimensions of the MDS solution

Dim	Stress	CV	RSS	P	$R^{2}$
1	0.36	.70 (.04)	65.92	32	0.55
2	0.16	.86 (.02)	12.14	64	0.79
3	0.11	.88 (.02)	6.36	96	0.86
4	0.09	.88 (.02)	4.10	128	0.90
5	0.07	.87 (.03)	2.66	160	0.93

*Note.* CV: average correlation (and standard deviation) between the predicted and the true pairwise distances over 500 repetitions of the cross-validation. RSS: Residual-sum-of-squares. P: Number of parameters of the MDS model. $R^2$: Explained variance of the empirical pairwise distances by the corresponding MDS model

### Cognitive modeling of judgment data (Steps 5 & 6 in Fig. 1)

#### Method

##### Materials

The stimuli were the same as in the similarity rating task. We used the maximum horizontal flight speed as the to-be-judged criterion, because it has a reasonable range of different values, people do not have much previous knowledge about the flight speeds of birds, and there are many different features of bird species which predict their flight speed (Alerstam, Rosén, Bäckman, Ericson, & Hellgren, 2007; Greenewalt, 1975; Hall & Heesy, 2011). For instance, the flight speed is related to size and mass of birds, but also phylogenetic effects play an important role, where species of the same group tend to fly at similar speeds (e.g., swans, geese & ducks fly faster than falcons, crows & songbirds, Alerstam, Rosén, Bäckman, Ericson, & Hellgren (2007)). We extracted the maximum flight speed values from scientific articles if possible (e.g., Johnson, Booms, DeCicco, & Douglas , 2017), or otherwise used the values from the original quartets game. Thus, participants judged the actual maximum flight speed of birds (as accurately as it could be determined), which in our sample of birds ranged from 24 km/h (great spotted woodpecker) to 125 km/h (mallard).

Before the experiment, we selected 12 from 32 possible birds as exemplars based on the MDS analysis. For different sets of 12 exemplar birds, we computed the prediction of a rule-only model and an exemplar-only model. We selected one exemplar set where the predictions of both models were different (as indicated by a high RMSE between the predictions of the rule-only model and the exemplar-only model), but still correlated highly with the external criterion value (i.e., the true maximum flight speed), and where the criterion values of the exemplar birds captured a high range of possible criterion values (the R script for this procedure can be found on the OSF). This procedure ensured that the RulEx-J model is able to separate rule-based and exemplar-based processes based on the responses of participants.

##### Design and procedure

The general design and procedure of this experiment was based on Pachur and Olsson (2012) and Trippas and Pachur (2019), where the experiments consisted of two main phases, a training phase and a testing phase. Depending on the condition, the task and the feedback during the training phase of the experiment were different for the participants. In the learning by comparison condition participants had to compare two stimuli in a trial and received only relative feedback but no feedback about the exact criterion values. In the direct criterion learning condition, participants had to classify one stimulus at a time and received feedback about the exact criterion values. The results of Pachur and Olsson (2012) and Trippas and Pachur (2019) showed that the direct criterion learning procedure elicited predominantly exemplar-based processing whereas learning by comparison lead to more rule-based judgments.

Using the same conditions, in our experiment participants in the learning by comparison condition were presented with pictures of two exemplar birds in each trial during the training phase. Participants were then asked to decide which of the two birds is the faster bird (i.e., has the higher maximum horizontal flight speed in km/h). After their choice, participants got feedback about the correct answer, as indicated by a blue border around the faster bird. One block in the training phase consisted of 65 trials, comprising all possible pairwise combinations of the 12 exemplar birds, except for one pair where the two birds had the same criterion value. Participants had to complete at least three training blocks, up to a maximum of seven training blocks. Participants could finish the training phase earlier if they meet a criterion of at least 85% correct responses after the third training block.

In the direct criterion learning condition, participants were presented with the picture of one out of the 12 exemplar birds in each trial of the training phase. They were asked to decide whether this bird is a fast bird (i.e., flight speed above 43 km/h, which is the median flight speed of the exemplar birds), or a slow bird (i.e., flight speed below 43 km/h). After each response, participants got feedback whether their answer was correct or not and what the exact maximum flight speed of the bird was. As in Trippas and Pachur (2019), participants were instructed before the start of the training phase to pay attention to this flight speed as it would be relevant in the testing phase. One training block consisted of all 12 exemplar birds. Participants had to complete at least ten training blocks, up to a maximum of 30 training blocks. Again, participants could finish the training phase earlier if they meet a criterion of at least 85% correct responses after the tenth training block.

The testing phase was the same for all participants. Participants were asked to judge the maximum flight speed of all 32 birds (12 old and 20 new birds). Before the start of the testing phase, we informed participants that the flight speeds can range from 24 km/h up to 125 km/h. In addition, we presented the Latin names of each bird below its picture throughout the experiment, so that participants could better remember and distinguish the birds.

At the end of the experiment, participants again answered demographic questions and were asked about their general knowledge of and interest in birds, rated on a scale from 1 (not much) to 5 (very much). They were also asked to indicate if their data should be used for data analysis or if it should be excluded (Aust, Diedenhofen, Ullrich, & Musch, 2013).

##### Hypothesis

Based on the original results of Pachur and Olsson (2012) and Trippas and Pachur (2019), we expected to find more rule-based processing in the learning by comparison condition, relative to the direct criterion learning condition.

##### Data analysis

As preregistered and as in the validation study, the judgment data were analyzed using the hierarchical Bayesian version of the *RulEx-J* model (Bröder, Gräf, & Kieslich, 2017; Izydorczyk, & Bröder , 2022) extended for continuous cues. The RulEx-J model proposes a continuous mixture between rule-based and exemplar-based processes in quantitative judgments. The model allows to measure the relative contribution of each type of process by using a mixing parameter $\alpha$, which measures the relative proportion of each process in the final judgment. According to the RulEx-J model, the actual final judgment $J$ is a weighted combination of both interim judgments, $J_R$ and $J_E$, from the respective rule- or exemplar-based processes:

1 $$\begin{aligned} J = \alpha \times J_R + (1-\alpha ) \times J_E, \end{aligned}$$

where the $\alpha$ parameter can range from 0 to 1, with larger values indicating more rule-based processing and smaller values indicating more exemplar-based processing. The rule-based process was modeled using the cue abstraction model (Juslin, Olsson, & Olsson, 2003) and the exemplar-based process by a simplified version of the generalized context model (Nosofsky, 1986) assuming equal cue weights (more information about the model and its implementation can be found in the Appendix. Based on our hypothesis that there will be more rule-based processing in the learning by comparison condition, we expect to find higher $\alpha$ values in the learning by comparison condition than in the direct criterion learning condition on average.

The hierarchical Bayesian implementation of the RulEx-J model proposed in Izydorczyk and Bröder (2022) directly incorporates the difference in the $\alpha$ parameter between two conditions via the parameter $\delta$ which reflects the differences of $\alpha$ between both conditions on a standardized scale. Hence, it reflects the effect size of the fixed effect between experimental conditions. For statistical inferences about group differences in $\alpha$, we can compute the Bayes factor based on the Savage-Dickey density ratio (SDDR, Vandekerckhove, Matzke, & Wagenmakers , 2015; Wagenmakers, Lodewyckx, Kuriyal, & Grasman , 2010) by computing the ratio of the prior density $p(\delta =0|\mathcal {H}_1)$ and posterior density $p(\delta =0|D,\mathcal {H}_1)$ at point $\delta$ = 0.^8^ Since we expected to find, on average, larger $\alpha$ values in the rule condition (i.e., $\delta>$ 0), we used only those MCMC samples that obeyed this order-restriction to calculate the densities, which corresponds to a one-sided test (Wagenmakers, Lodewyckx, Kuriyal, & Grasman, 2010). The resulting Bayes factor of this ratio $BF_{10} = \frac{p(\delta =0|\mathcal {H}_1)}{p(\delta =0|D,\mathcal {H}_1)}$ indicates the relative evidence for $\mathcal {H}_1$ (i.e., $\delta>$ 0, average $\alpha$ is higher in the learning by comparison condition) compared to $\mathcal {H}_0$ (i.e., $\delta$ = 0, no difference between conditions, Kass & Raftery , 1995; Morey, Romeijn, & Rouder , 2016; Vandekerckhove, Matzke, & Wagenmakers , 2015). We used JAGS (Plummer, 2003) interfaced with R using the runjags package (Denwood, 2016) to fit the model.

We ran 4 chains of 150,000 samples each, collected after 30,000 burn-in samples were discarded, 30,000 adaptive iterations, and thinning by recording every 30th sample. The convergence of the chains was checked by visual inspection and the standard $\hat{R}$ statistic ($\hat{R}$ 1.01, Gelman & Rubin 1992). The R script, the JAGS model with the prior specifications, the MCMC traces, and the results files can be found in the OSF of this project.

##### Participants

In total, we collected data from $N$ = 80 participants through Prolific Academic. We excluded $n$ = 1 participant because he indicated that his data should not be used for analysis and $n$ = 1 participant who was not fluent in English.^9^ The final sample consisted of $N$ = 78 participants (56.41 % female) with an average age of 41.08 ($SD$ = 14.17). Out of these $N$ = 78 participants, $n$ = 39 were in the leaning by comparison condition and $n$ = 39 in the direct criterion learning condition.

#### Results

First, we examine the performance of the participants during the test phase, and subsequently, we shift our focus towards the cognitive modeling results. As preregistered, our main analysis will focus on the difference in rule- and exemplar-based processing between the two conditions. In addition, we will report results of a formal model comparison between the RulEx-J model and a pure rule-based and a pure exemplar-based model, as well as some posterior predictive checks. The analysis of the performance during the training phase is available in the supplementary material provided online on the OSF.

##### Performance

**Fig. 4:**
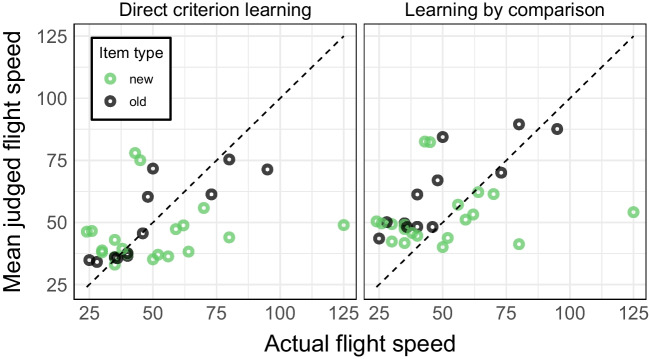
Mean judgments for each of the *K* = 32 bird, separately for the two training conditions

Fig. 4 shows the mean estimates for old and new stimuli, separately for the two training conditions. The accuracy in the testing phase was defined as the root-mean-square error (RMSE, smaller values indicate less error or higher accuracy) between birds’ estimated and actual flight speed. Accuracy was higher (i.e., RMSE was lower) in the direct criterion learning condition ($M$ = 22.54, $SD$ = 8.04) compared to the learning by comparison condition ($M$ = 30.38, $SD$ = 6.90), $F(1, 76) = 40.31$, $p <.001$, $\hat{\eta }^2_G =.272$. In addition, participants had a higher accuracy for old ($M$ = 23.17, $SD$ = 10.01) than for new birds ($M$ = 29.75, $SD$ = 4.61), $F(1, 76) = 67.18$, $p <.001$, $\hat{\eta }^2_G =.208$. Furthermore, the difference in performance between old-new items was also different between the conditions, as indicated by the significant interaction ($F(1, 76) = 21.06$, $p <.001$, $\hat{\eta }^2_G =.076$) where the difference in accuracy between old and new items was smaller in the learning by comparison condition ($M_{\Delta }$ = 2.89) than in the direct criterion learning condition ($M_{\Delta }$ = 10.25). These pattern of results are similar to the performance patterns found in Experiment 3 of Pachur and Olsson (2012), which included the same manipulations of the learning task and a non-linear environment.

**Cognitive modeling**

##### Difference in processing between conditions

As predicted, the SDDR Bayes Factor indicated extreme evidence for our hypothesis that there is more rule-based processing for participants in the learning by comparison condition compared to the direct criterion learning condition ($BF_{10}$ > 1000).^10^ The corresponding posterior distribution of the effect size parameter $\delta$ had a mean of 2.31 ($SD$ = 0.55) with a 95%-HDI ranging from 1.31 to 3.49. Correspondingly, the $\alpha$ parameter of the RulEx-J model was higher in the learning by comparison condition ($M$ =.25, 95%-HDI [.20,.31]) compared to the direct criterion learning condition ($M$ =.15, 95%-HDI [.12,.19]), as indicated by the probit transformed group-level parameters $\mu _{\alpha , j = \{1,2\}}$ (see the Appendix).

##### Model comparison

Based on the relative strength of evidence as indicated by the log(Bayes Factors) shown in Table 2, the RulEx-J model did better account for people’s judgments than the corresponding sub-modules (i.e., pure rule-based processing or pure exemplar-based processing, see Appendix). In addition, the exemplar-based model did also better account for the data than the rule-based model, even in the learning by comparison condition. The Bayes Factors were calculated with bridge sampling (Gronau et al., 2017; Gronau, Singmann, & Wagenmakers, 2018).

**Table 2:** Model comparison using log(BF)

Condition	$\mathcal {M}_1$: RulEx-J	$\mathcal {M}_1$: Exemplar	vs. $\mathcal {M}_0$
DCL	118.96		Exemplar
	314.72	195.76	Rule
LBC	289.82		Exemplar
	307.10	17.29	Rule

*Note.* Positive values of log(Bayes Factor) indicating evidence in favor of $\mathcal {M}_1$ and negative values indicating evidence in favor of $\mathcal {M}_0$. DCL = direct criterion learning, LBC = learning by comparison

##### Posterior predictive checks

As indicated by the RMSE and correlation, the means of the posterior predictive distributions per trial reasonably well reflected participants’ actual judgments on average, but not for each participant, in the learning by comparison (RMSE: $M$ = 13.96, $SD$ = 5.75, *range*: 2.21 - 29.14; $r$: $M$ =.66, $SD$ =.23, *range*: -.25 -.95) and the direct criterion learning condition (RMSE: $M$ = 11.58, $SD$ = 5.85, *range*: 2.75 - 35.05; $r$: $M$ =.72, $SD$ =.19, *range*:.06 -.96).

## General discussion

In two studies we demonstrated how multidimensional scaling can be used to generate cues and cue values of naturalistic stimuli, which then can be used in cognitive models of numerical judgments. The procedure presented in this work is based on the recent work about classification learning in high-dimensional natural-science category domains of Nosofsky and colleagues (Nosofsky, Sanders, Meagher, & Douglas , 2018, 2020; Nosofsky, Sanders, & McDaniel, 2018; Nosofsky, Sanders, Zhu, & McDaniel, 2019), but extended to continuous judgments. In a validation study, we showed that the reconstructed dimensions based on pairwise similarity ratings and the results of a subsequent analysis of judgment data were virtually identical to the original results using pre-defined cues. In a second study, we were able to replicated the results of previous experiments reported in Pachur and Olsson (2012) and Trippas and Pachur (2019) using complex naturalistic images with an unknown cue structure. In the following, we discuss some important implications and limitations of our work.

### Comparison to results of laboratory studies

There are several differences between the original studies of Pachur and Olsson (2012) and Trippas and Pachur (2019) and our replication study using complex stimuli. For instance, the differences in the learning environment’s complexity, the number of stimuli, and the scale and distribution of criterion values. Nevertheless, our computational modeling results reproduce the general finding that there is more rule-based processing in the learning by comparison condition than in the direct criterion learning condition. Furthermore, the performance of participants in the testing phase and the general observed judgment patterns shown in Fig. 4 are also in line with previous findings. As in Experiment 3 in Pachur and Olsson (2012), which included the same manipulations of the learning task and a non-linear environment (as is case for the flight speed of birds), participants trained with learning by comparison provided less accurate judgments than those trained with direct criterion learning. However, it should be noted that in our study participants seemed to be less able to transfer their learned knowledge to new stimuli than in the original studies of Pachur and Olsson (2012) and Trippas and Pachur (2019). Furthermore, the model comparison results and the general low levels of $\alpha$ suggest that even though there was *more* rule-based processing in the learning by comparison condition than in the direct criterion learning condition, participants in both conditions predominantly relied on an exemplar-based strategy to make their judgments. This is in line with an abundance of empirical findings showing that participants rely more on exemplar-based processing in a non-linear environment or in general when it is difficult to abstract a linear additive rule (e.g., Hoffmann, von Helversen, & Rieskamp , 2016; Juslin, Karlsson, & Olsson , 2008; Platzer & Bröder, 2013; von Helversen, Karlsson, Mata, & Wilke , 2013). Although participants in the learning by comparison condition never got feedback about the actual criterion values needed for exemplar-processing, they probably developed some (erroneous) representation of the criterion values based on the scale of the criterion and the learned rank order.

### Quality of extracted cues

In order to model participants’ judgments with the assumed rule- and exemplar-based models we used MDS analysis based on similarity ratings to extract the necessary cues. However, the MDS-based cues people use to make their similarity ratings do not necessarily have to correspond to the features which are actually important for predicting or describing the criterion in the environment. In our case however, the extracted cues seem to be actually good predictors of the flight speed of birds. According to Alerstam, Rosén, Bäckman, Ericson and Hellgren (2007), important predictors for the flight speed of birds are phylogenetic group (e.g., Swans/geese/ducks vs. falcons/ crows/songbirds), wing loading, the aspect ratio of wingspan and wing area, and the body mass, which is highly correlated with wing load. The phylogenetic group and wing loading are the most important predictors. In our study, the first and second dimension in the the MDS space shown in Fig. 3 might correspond to size/mass and some form of crude phylogenetic categorization. Using all three dimensions in a linear model they explain 55.32 of the variance (as indicated by the adjusted $R^2$) in flight speed when no interactions are allowed (i.e., a linear additive model as assumed by the rule-based process). More details can be found in the supplement. Nevertheless, there might be cases where the extracted cues are not predictive of the actual criterion or that people might base their similarity judgments on different cues than when making judgments about a some other criterion. Thus, better cues for modeling people’s judgments might be obtained by asking for similarity ratings regarding specific criteria, rather than asking for a general similarity rating between stimuli. For instance, when asked about the general similarity between sports people might rely on cues such as individual vs. team sport, ball vs. no ball, or on land vs. on water. But when asked about the similarity between sports regarding players’ income potential, people might use cues such as screen time on TV or number of fans. In addition, one limitation of our procedure was to use average similarity ratings to extract the cues, since every participant only rated a manageable subset (25%) of all possible 496 stimulus pairs. While the resulting multidimensional space adequately describes this averaged data, it may not capture individual participants’ representations (Ashby, Maddox, & Lee, 1994; Estes, 1956). Thus, computing individual-level MDS solutions could also lead to more individually valid cues overall and may increase the model’s ability to account for individual participants’ judgments.

### Modeling

So far, we used the RulEx-J model to model participants’ judgments which assumes a continuous blending between a rule-based process and an exemplar-based process (Bröder, Gräf, & Kieslich, 2017; Izydorczyk, & Bröder , 2022), which we modelled using the cue abstraction model (Juslin, Olsson, & Olsson, 2003) and a simplified version of the generalized context model (Nosofsky, 1986) assuming equal cue weights. Like all models, the RulEx-J model is so far intended as a pragmatic tool to measure the mixture between rule- and exemplar-based processes (which was the main focus of our hypothesis) and thus might not describe the actual cognitive processes that lead to a judgment. As of yet, we did not test whether other models, such as the CX-COM model (Albrecht, Hoffmann, Pleskac, Rieskamp, & von Helversen, 2019) or the mapping model (von Helversen & Rieskamp, 2008), are better able to capture participants’ judgments in these tasks, or if using different sub-models as characterization of the rule- and exemplar-based processes in the RulEx-J model would lead to different results. Finally, although our model checks indicate a reasonably good correspondence between model predictions and actual judgments for most participants, there are some individuals for whom this correspondence is not observed. Consistent with the original experiments by Trippas and Pachur (2019) and Pachur and Olsson (2012), we did not use any performance-based inclusion criteria, such as accuracy in the training phase or in the final testing phase. Given the higher difficulty of our task, excluding participants who did not perform well in either the training or the testing phase may improve model fit and might be useful to consider for future studies.

**Fig. 5:**
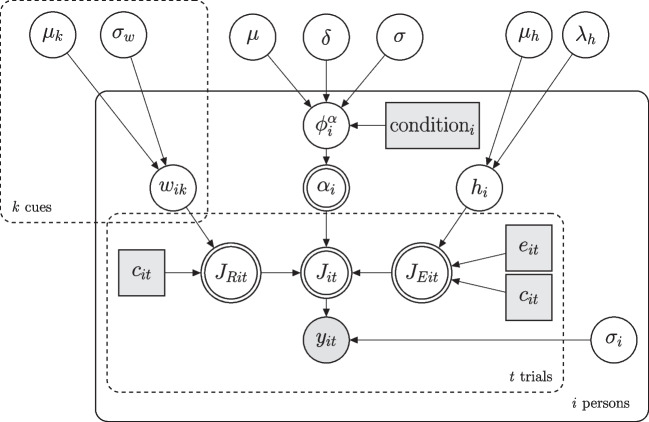
Graphical model of the RulEx-J model for continuous cues with between condition difference in $\alpha$

### Conclusion

Building on earlier and recent categorization research (Nosofsky , 1992; Nosofsky, Sanders, Meagher, & Douglas , 2018; Nosofsky, Sanders, & McDaniel, 2018; Shin & Nosofsky , 1992) we present a method which makes it possible to apply well tested and established models of numerical judgment processes to realistic rather than artificial stimuli. In the future, this may be useful in order to use computational cognitive models, which were so far only used inside the laboratory, to investigate real life estimation or judgment problems, for instance, how people estimate the amounts of sugar in food items or carbon footprints of consumer goods.

## Data Availability

All scripts, results, experiment files, the preregistration of the judgment experiment in Study 2, the data, and stimuli are available at the Open Science Framework (OSF, https://osf.io/yaudb/). This paper was written in RMarkdown using the papaja package (Aust & Barth, 2020), which includes all code for analyses and figures. The RMarkdown file can also be found on the OSF.

## RulEx-J Model for continuous cues

The graphical model of the hierarchical Bayesian RulEx-J model is depicted in Fig. 5. We use the notation of Lee (2008), in which observed variables (i.e., the data) are shown as shaded nodes and unobserved variables (i.e., model parameters to be inferred) are shown as unshaded nodes. Discrete variables are indicated by square nodes and continuous variables are indicated by circular nodes. Unobserved stochastic variables are indicated by single-bordered nodes, and unobserved deterministic variables are indicated by double-bordered nodes.

The RulEx-J model assumes that rule-based and exemplar-based processes work in parallel and that a judgment $y$ of a person $i$ in a trial $t$ is a mixture of both distinct processes (Bröder, Gräf, & Kieslich, 2017; Izydorczyk, & Bröder , 2022). When a probe (i.e., a stimulus which has to be judged) is presented to a person, it will be processes by an exemplar module $E$ and a rule module $R$, each making their own distinct judgments ($J_E$ and $J_R$). According to the RulEx-J model, the final judgment is then a mixture of the judgments of both modules:

A1 $$\begin{aligned} y = \alpha \times J_R + (1-\alpha ) \times J_E \end{aligned}$$

where $\alpha$ is the mixture parameter, which measures the relative contribution of subprocess. We will first describe how the rule and exemplar modules are defined and then how the difference between conditions in $\alpha$ is implemented in the model.

### The rule module

Rule-based process models assume that people combine and integrate cue information according to some abstracted rule to make a judgment (Juslin, Olsson, & Olsson, 2003). The rule, according to which the information of multiple cues is integrated, is often assumed to be a linear additive function (Einhorn, Kleinmuntz & Kleinmuntz, 1979; Hoffmann, von Helversen, & Rieskamp , 2019; Juslin, Karlsson, & Olsson , 2008). Thus, the judgment $J_R$ of the rule module is generated by:

A2 $$\begin{aligned} J_R = w_0 + \sum _{k = 1}^{n_k} \textrm{cue}_k \times w_k \end{aligned}$$

where $J_R$ is the judged criterion of an object $p$ (the probe) based on the intercept $w_0$ and the cue weights $w_k$ for the corresponding $n_k$ cues. This rule-based model, sometimes referred to as *cue abstraction model* (Juslin, Olsson, & Olsson, 2003), is quite flexible and does not necessarily imply a compensatory processing of all cues, but can also mimic simpler strategies or heuristics focusing on one or only few cues by choosing appropriate (zero) cue weights (see Bröder , 2000). Each cue weight $w_k$ comes from a normal distribution with mean $\mu _k$ and a common standard deviation $\sigma _w$, with the following hyperpriors:

A3 $$\begin{aligned} w_k&\sim \textrm{Normal}(\mu _k,\sigma _w) \end{aligned}$$

A4 $$\begin{aligned} \mu _k&\sim \textrm{Normal}(0,5) \end{aligned}$$

A5 $$\begin{aligned} \sigma _w&\sim \textrm{Exp}(0.5) \end{aligned}$$

### The exemplar module

The exemplar module is defined based on the *generalized context model* (GCM, Nosofsky , 1986; 2011) extended to numerical judgments. The GCM as well as related models used in the multiple-cue judgment literature, implicitly assume an integrative retrieval of exemplars where all previously encountered exemplars and their criterion values are retrieved from memory and then integrated into the final judgment (cf., Albrecht, Hoffmann, Pleskac, Rieskamp, & von Helversen, 2019; Nosofsky & Palmeri , 1997). The similarity of the probe to each of the exemplars acts as a weight in the integration of all exemplar criterion values into the final judgment. More similar exemplars receive more weight and thus their criterion values have a higher impact on the final judgment (Medin & Schaffer, 1978; Estes, 1994)). Formally, in the GCM the distance between the probe $p$ in a given trial and a given exemplars $e$ is computed as:

A6 $$\begin{aligned} d_{pe} = \sqrt{\sum _{k = 1}^{n_k} w_k \times (p_k - e_k)^2 } \end{aligned}$$

which is the root of the summed squared difference between the cue values of the probe and the exemplar on each cue $k$. The weights $w_k$ are assumed to sum to 1 and weigh how much attention each cue or dimension receives. However, based on previous results (Hoffmann, von Helversen, & Rieskamp, 2013, 2014, 2016; von Helversen & Rieskamp, 2008) we omitted the $w_k$ parameters and thus the distance of each exemplar to the probe is computed as:

A7 $$\begin{aligned} d_{pe} = \sqrt{\sum _{k = 1}^{n_k} (p_k - e_k)^2 } \end{aligned}$$

The distance gets converted into a similarity according to:

A8 $$\begin{aligned} s_{pe} = e^{-hd_{pe}} \end{aligned}$$

where $h$ is the sensitivity parameter, which reflects the rate at which similarity declines with distance. Smaller sensitivity parameters indicate that similarity declines less with distance. The sensitivity parameters of all participants are assumed to follow truncated normal distribution with mean $\mu _h$, standard deviation $\sigma _h$ and a lower bound of 0, with the following hyperpriors:

A9 $$\begin{aligned} h&\sim \textrm{Normal}_{[0,]}(mu_h,\sigma _h) \end{aligned}$$

A10 $$\begin{aligned} \mu _h&\sim \textrm{Normal}(0,2) \end{aligned}$$

A11 $$\begin{aligned} \sigma _h&\sim \textrm{Exp}(1) \end{aligned}$$

The final judgment $J_E$ of the probe is then computed by weighting the criterion value $c$ of each exemplar by the similarity of the exemplar to the probe, summing everything together, and dividing everything with the summed similarity to get the scale correct (Elliott & Anderson , 1995; Juslin, Olsson, & Olsson, 2003; Juslin & Persson , 2002).

A12 $$\begin{aligned} J_E = \frac{\sum _{e=1}^{n_e}s_{pe} \times \textrm{criterion}_e}{\sum _{e=1}^{n_e}s_{pe}} \end{aligned}$$

Note, that the formulation of the exemplar module of the RulEx-J model here is different from the original formulations in Bröder, Gräf, and Kieslich (2017) and Izydorczyk and Bröder (2022), which used the *Context Model* (Medin & Schaffer, 1978) instead of the GCM. However, since the cues extracted by the multidimensional scaling analysis are continuous rather than binary as in Bröder, Gräf, and Kieslich (2017), we used the GCM here.

### The blending

The predictions of both modules are then weighted according to the mixture parameter $\alpha$ as stated in Eq. 1. The $\alpha$ parameter of each person $i$ comes from one of two potentially different overarching Gaussian distributions for the *lbc* and the *dcl* condition. The means of these distributions are expressed in terms of a parameter representing the overall mean ($\mu _{0}$) and a parameter representing the difference between the means for the two conditions ($\delta$):

A13 $$\begin{aligned} \mu _{\alpha , j=1}&= \mu _{0} + \frac{1}{2} (\delta \times \sigma _{\alpha }) \end{aligned}$$

A14 $$\begin{aligned} \mu _{\alpha , j=2}&= \mu _{0} - \frac{1}{2} (\delta \times \sigma _{\alpha }) \end{aligned}$$

where parameter $\mu _{0}$ reflects the overall $\alpha$ mean on the real scale. The parameter $\delta$ reflects the differences between both conditions on a standardized scale and hence, it reflects the effect size of the fixed effect between experimental conditions. The $\alpha$ value of each person $i$ on the real scale ranging from $-\infty$ to $\infty$ ($\alpha _{real_i}$) is then drawn from a normal distribution with a mean depending on the condition of the person with $\mu _{\alpha , j=1}$ for the *learning by comparison* condition and $\mu _{\alpha , j=2}$ for the *direct criterion learning* condition. To get $\alpha$, the $\alpha _{\textrm{real}_i}$ is then probit transformed to make sure the values are on the scale from 0 to 1.

A15 $$\begin{aligned} \alpha _{\textrm{real}_i}&\sim \textrm{Normal}(\mu _{\alpha j},\sigma _\alpha )\end{aligned}$$

A16 $$\begin{aligned} \alpha _i&= \Phi (\alpha _{\textrm{real}_i}) \end{aligned}$$

We used the following hyperpriors:

A17 $$\begin{aligned} \mu _{0}&\sim \textrm{Normal}(0,1) \end{aligned}$$

A18 $$\begin{aligned} \delta&\sim \textrm{Normal}(0,1) \end{aligned}$$

A19 $$\begin{aligned} \sigma _{\alpha }&\sim \textrm{Exp}(0.5) \end{aligned}$$

## Footnotes

1 For a more detailed account of the reasons why we favour the “classic” MDS approach over the DNN approach and additional materials (e.g., correlation of participants’ similarity ratings the predicted similarities based on features extracted from a neural network) can be found in the supplement on the OSF.

2 Please note that when we refer to “generating cues,” we are not necessarily referring to the creation of objective features that completely define an object (although this might be possible with simple stimuli as used in Study 1), but rather identifying cues which are psychological dimensions (not necessarily objective or nameable features) used to represent these objects in psychological space.

3 The data are publicly available on the OSF (https://osf.io/yaudb/)

4 Because of licencing issues, the images of nine birds on the OSF are different from the original images used in the experiment. However, the new pictures are almost identical to the original images as we selected the new pictures to match the original pictures in posture, lighting, and other factors as closely as possible. All images are now published under a CC BY-SA 4.0 license on the OSF.

5 The subsequent reported results do not change, when these participants were included.

6 For instance, the average similarity of Pair 1 was 2.38. The normalized value then is $\frac{(2.38--1)}{(7-1)} =.23$ and the corresponding normalized dissimilarity $1-.23 =.77$. The normalization was used in order to calculate the BIC for determining the dimensionality of the MDS space as proposed by Lee (2001). The reported results do not change substantially if the normalization step is skipped.

7 The R code to run the cross-validation is available in the MDShelper package on Github https://github.com/dizyd/MDShelper

8 The density of the posterior distribution was computed with the dlogspline function in the polspline package in R (Kooperberg, 2020).

9 This exclusion step was not preregistered, but the subsequent reported results do not change, when this participant is included.

10 The results do not substantially change when only the responses to the new stimuli in the testing phase are used as data or when the learning and direct recall of exemplars criterion values is incorporated in the model using a latent-mixture extension (Izydorczyk, & Bröder, 2021; Zeigenfuse & Lee, 2010) For details see the online supplement.
